# Learning health system strategies in the AI era

**DOI:** 10.1038/s44401-025-00029-0

**Published:** 2025-06-17

**Authors:** Peter A. D. Steel, Gabriel Wardi, Robert A. Harrington, Christopher A. Longhurst

**Affiliations:** 1 Department of Emergency Medicine, NewYork-Presbyterian/Weill Cornell Medicine, New York, NY USA; 2 Department of Emergency Medicine, University of California at San Diego, San Diego, CA USA; 3 Department of Internal Medicine, University of California at San Diego, San Diego, CA USA; 4 Stephen and Suzanne Weiss Dean, Weill Cornell Medicine, New York, NY USA; 5 San Diego School of Medicine, University of California, San Diego, CA USA

**Keywords:** Information systems and information technology, Health services

## Abstract

The learning health system (LHS) offers a framework to accelerate evidence generation and care improvement, yet widespread adoption remains limited. In this perspective, we explore strategies to operationalize the LHS in the era of artificial intelligence, including biomedical informatics and health information technology integration, workforce development, quality improvement, and data governance. We highlight promising institutional models and propose policy, educational, and financial reforms to support scalable, value-driven innovation in increasingly complex and resource-constrained health systems.

## Introduction

The learning health system (LHS) can be defined as the infrastructure, diverse stakeholders, and culture required to integrate continuous cycles of evidence-based care model improvement and innovation^1^. This cyclical process consists of data collection and analysis, generation of new knowledge, and subsequent translation of this knowledge into interventions designed to improve healthcare quality and value^2^. The LHS concept has been endorsed by the Joint Commission, Agency for Healthcare Research and Quality (AHRQ), the National Academy of Medicine, and the World Health Organization^3–5^. Consensus of the model’s potential to transform health systems has been accelerated by mandates for greater efficiency and higher-quality care in the setting of growing fiscal constraints. But while the LHS ecosystem and its requisite competencies have been well described^1,6–10^, multidimensional limitations to cultivating this complex adaptive system persist, including siloed data, lack of adaptive governance, misaligned incentives, underdeveloped workforce competencies and cultural resistance to change^11^. This *Perspective* argues that to fully realize the transformative potential of artificial intelligence (AI) in health care, and for the long-standing translational research gap to be closed, LHS principles must evolve from aspirational goals to a consensus strategy in US health systems. We describe how the challenges facing AI implementation and LHS development frequently overlap and explore how AI may serve to accelerate the LHS, offer examples of health systems that have successfully adopted LHS principles, highlight strategies to address common challenges in the era of AI, review current and future approaches to workforce education, and conclude with recommendations for how the LHS might successfully evolve in the AI era despite increasing fiscal constraints.

## Ambidextrous health information technology, biomedical informatics leadership

Widespread adoption of the electronic health record (EHR) marked the beginning of the digital age of health care. Subsequent advances in big data^12^, digital care^13^, and AI^14^ have created opportunities for alignment between health information technology (HIT) and biomedical informatics (BMI). HIT encompasses the hardware, software, infrastructure design, and implementation expertise necessary to manage and protect clinical and administrative data across a health system. This includes software selection, contract negotiation, systems integration and testing, and user training—all aimed at delivering enterprise-wide, reliable, cost-effective, and secure digital solutions in support of operational stability and strategic goals. In contrast, BMI is an interdisciplinary scientific field focused on the effective use of biomedical data, information, and technology to improve human health. BMI includes disciplines such as data science, natural language processing, predictive modeling, and implementation science, and is often directed at solving complex problems across people, processes, and technologies^15^. When aligned, HIT and BMI enable organizations to balance the demands of operational reliability with the need for continuous learning and innovation. When aligned, HIT and BMI enable organizations to balance the requirements for operational reliability and effectiveness with needs for change, growth, and discovery^16,17^. Such *ambidextrous leadership*—the optimization of current operations while simultaneously driving novel solutions to challenges and innovation—has been shown to improve organizational performance in both healthcare and technology industries^18,19^.

The COVID-19 pandemic accelerated many health systems’ adoption of LHS values and demonstrated the impact from strategic allocation of cross-disciplinary organizational resources to support time-sensitive quality and operational priorities, as well as high-value research^20–27^. Productive collaboration between HIT and BMI efficiently generated knowledge for time-sensitive clinical and operations decision-making; and strategic implementation of digital health innovations at scale. Examples included the rapid development of capacity-demand prediction dashboards through HIT–BMI collaboration, telehealth-supported direct and remote patient monitoring care models in a New York City health system challenged with inpatient capacity; basic laboratory test result-based predictions of Covid infection, pneumonia, and clinical deterioration; and the rapid implementation of a EHR-integrated employee vaccination superstation^28–31^. Academic institutions reporting maturation in this *operationalized synergy* of HIT and BMI describe the stakeholder engagement, relationship building, and collaborative, matrixed leadership required to achieve significant gains, including the pragmatic timelines for such transformation^8,15,27,28,32^. Another urgent priority was the democratization of data access for rapid-cycle testing of quality improvement processes by limiting regulatory barriers without compromising research ethics, patient privacy, and consent standards, which a few academic health systems have described^28,33^.

Limitations to high-performing routine HIT systems, such as issues with data quality, missingness, and bias; data access and siloed data from disparate sources; errors in data production; lack of standardized data production processes; and inadequate interoperability, all continue to conspire against the broad implementation of an LHS model^34,35^. Data harmonization techniques have been described in detail, as it has the importance of universally recognized data standards^34,36,37^. Recent studies have demonstrated that large language models (LLMs) are capable of executing data extraction, preprocessing, and foundational analytic tasks with increasing reliability^38–41^. A future with automated or semi-automated data harmonization could be transformative, significantly reducing barriers to data acquisition and standardization and enabling researchers to devote greater effort towards higher-order analytics and model development. However, the implementation of LLMs for such foundational tasks will require strategies to mitigate hallucinations, ensure pipeline transparency and auditability, and address challenges related to reproducibility and regulatory compliance^42^. While such foundational progress continues, rapid growth in digital health innovation and the increasing demand for health data have brought new challenges. These include capacity limitations and high costs for data storage and analysis; gaps in the translational science expertise needed to ensure diverse BMI clinical interventions (e.g., AI-based clinical decision support, guideline delivery systems, population health management platforms and dashboards) are effectively integrated into clinical workflows and clinician practice to yield the desired impact^43–47^, as well as the capacity and leadership for functional governance of these complex systems^48–50^.

## Research and quality in the AI era of the LHS: two sides of the same coin?

To date the explosion of healthcare AI research has predominantly centered on the development and validation of models using curated data sets, efforts that have demonstrated the potential of AI rather than its real-world impact^46,51–53^. In contrast, relatively few AI tools have been implemented into clinical workflows and rigorously evaluated for their effect on patient-centered outcomes or cost-effectiveness. Such implementation efforts are substantially more ambitious endeavors, requiring high-quality, interoperable real-time data, substantial computational power, and the technical expertise to enable seamless EHR integration. Moreover, the risk of distributional shift and data drift poses a threat to the sustained performance of deployed models, necessitating adaptive or *continuous learning* systems. These capabilities further increase complexity to the informatics infrastructure and technical expertise required to support them^54–56^. The need for rapidly accessible, interoperable data, alongside robust data management and governance frameworks, has long been championed by healthcare researchers and research ethicists, yet remains largely aspirational across most health systems^37^. However, growing recognition that high-performing HIT–BMI capabilities are foundational to the safe and effective clinical implementation of adaptive AI algorithms underscores a critical insight: data readiness is the essential first step toward AI readiness, closely followed by clinician readiness^57–59^. The promise of AI enhancing care quality and operational efficiency may be the tipping point for health systems to regard investments in data infrastructure and specialized workforce development as strategic imperatives, particularly for the deployment of locally developed algorithms in academic medical centers^60,61^.

The benefits afforded to the LHS are self-evident: the rapid acquisition of data and prompt analysis of care delivery gaps, along with the evaluation of interventions designed to close them. Notable examples of centralized health system network data repositories—Patient-Centered Outcomes Research Institute’s PCORnet and INSIGHT clinical research network, and University of California’s Center for Data-driven Insights and Innovation (CDI2)—demonstrate the potential to advance care quality, enhance health system operations, and accelerate clinical research^62–64^. Yet, despite the downstream opportunities such investments present, many health systems face persistent challenges in generating meaningful translational research. Contributing factors include prohibitive costs for the requisite infrastructure (particularly local development of AI models), concerns regarding data privacy and sharing, inflexible institutional review (IRBs) processes, and competing organizational priorities. The traditional model of research within most health systems has been heavily dependent on funding from large federal agencies (e.g., the National Institutes of Health), with a predominant focus on basic science^65^. While this emphasis has advanced critical foundational knowledge, it may have constrained the translation of discoveries into clinical impact. This paradigm is now under pressure from shifting federal research priorities away from the historic investments in basic science, including reductions in both direct funding and indirect costs support^65–67^. Amid these challenges may emerge the opportunity for health systems to realign their strategic research capabilities with evolving funding priorities, potentially strengthening capacity for translational and clinical research that advances LHS objectives.

Pre-clinical *silent* trials of clinical decision support (CDS) tools, run in the background without influencing clinical care, have been endorsed by ethical AI implementation frameworks. These trials have been shown to improve model performance and may assist with successful downstream implementation^68,69^. Integration of CDS tools into the EHR enables prospective, quality improvement-focused validation, including assessments of data drift, bias, and feasibility through mixed-methods studies, the results of which can inform downstream CDS design refinements prior to the safe execution of clinical studies^69^. Similarly, rapid cycle comparative effectiveness trials of quality improvement interventions have been facilitated through total EHR-integration of automated A/B and randomized testing methods^70–72^. These approaches can guide critical health system decisions regarding intervention impact and support iterative user-centered design, closing the traditional gap between clinical operations and the cost and expediency of rigorous scientific evaluation.

The principles of the LHS not only provide a foundation for generating high-quality research but also offer a natural pathway to an alternative approach to rapidly testing interventions: quality improvement. The National Academy of Medicine defines *Quality* as the “degree to which health services for individuals and populations increase the likelihood of desired health outcomes and are consistent with current professional knowledge”. Continual quality improvement is a core concept of the LHS. A foundational pillar in optimizing QI outcomes is the democratization of data access for rapid cycle testing of quality improvement processes by limiting regulatory barriers without compromising research ethics, patient privacy, and consent standards. The UC San Diego Health System developed a multidisciplinary stakeholder group approach (Align and Coordinate Quality Improvement, Research, and Evaluation–ACQUIRE), including members of the IRB, to expedite quality improvement and health sciences research that is aligned with health system priorities^33^. This approach provides the foundation for meaningful quality initiatives, some of which have had a profound impact on patient-centered outcomes^73,74^. Other health systems have described similar approaches with meaningful improvements in quality, for example, the impact of large-scale, randomized patient notification promoting influenza vaccination compliance to optimize messaging strategies^75,76^. New York University Langone Health has also championed *randomized* quality initiatives (e.g., strategies to improve smoking cession, minimizing missed appointments) and reports that the savings from these interventions more than paid for the operational costs for QI programmatic oversight needed to run the program overseeing these quality initiatives^70^.

## Education and workforce development

Novel frameworks and tools have been used to identify common barriers to implementing the LHS^77^, including inadequate stakeholder understanding of LHS principles and processes, and insufficient training for healthcare professionals (HCP) to effectively fulfill their collaborative roles within the model^11,78^. The proliferation of Master of Business Administration (MBA) programs in healthcare, often linked to the recognition of systemic challenges in U.S. healthcare, emphasizes management training^79^. The value of MBA education in advancing population health outcomes remains unclear, with studies demonstrating diverse motivations among healthcare MBA students^80–83^. Similarly, traditional graduate and post-graduate research training programs have not been primarily designed to develop the attributes and skills required for research within an LHS framework. Despite the absence of an established standard for US healthcare workforce education in health system improvement science (or their roles in it), a diverse range of educational programs serve to cultivate expertise aligned with LMH principles.

Since 2018, the Patient Centered Outcomes Research Institute and the AHRQ have jointly supported the development of LHS Researcher Training Programs and LHS Centers of Excellence. Their explicit goal has been to train researchers in the core competencies necessary for the collaborative generation of new knowledge that can be rapidly implemented to improve the quality of care, patient outcomes, and health system performance^84^. In addition to traditional research methods, these competencies include systems science; ethics of research and implementation in health systems; improvement and implementation science; engagement, leadership, and research management^6,85^. Similarly, curricula of Accreditation Council for Graduate Medical Education (ACGME)—accredited clinical fellowship programs in both Clinical Informatics (a subspecialty recognized by ABMS) and Healthcare Administration, Leadership, and Management have included many comparable competencies aligned with LHS concepts^86,87^. Further, some contemporary clinical informatics master’s programs have begun to incorporate focused, multidisciplinary management training, aimed to equip BMI leaders with the requisite leadership skills to successfully design and implement novel healthcare technologies into clinical practice^88–90^.

Other LHS educational programs have been described for PhD students^91^, clinician trainees and clinicians^92–95^, as well as for interdisciplinary professionals^96,97^; including LHS postgraduate certificate and degree programs at US centers of excellence^98,99^. Importantly, such changes can be easily added within existing training programs with minimal additional cost. For example, surgical residents at the University of California, San Diego may gain experience with LHS principles during protected research time. Skills acquired through this training have been instrumental in improving completed operative consent documentation, adherence to duty hour regulations, and crisis management^25,100,101^. However, formal evaluation of these training programs is limited. Future research is needed to examine their long-term impact on trainee career trajectories, to develop training strategies that engage non-specialist frontline HCPs, and to evaluate the broader effects of these interventions on staff engagement, wellbeing, and workforce retention^96,102–105^.

The digital age of healthcare has introduced new challenges well beyond the longstanding fragmentation and operational complexity of the U.S. health system. Clinicians face a growing crisis of information overload, compounded by traditional educational models that emphasize biological knowledge mastery, clinical information acquisition, and task completion^106^. The anticipated disruption of knowledge-based tasks by AI presents an opportunity to evolve medical training by integrating traditional clinical reasoning with computational expertise. Recent proposals have called for a radical redesign of medical curricula, prioritizing higher-order meta-cognitive skills such as knowledge management and contextualized synthesis, as well as problem solving, and discovery^107^. Curricular reform should also address the practical application of digital technologies; foundational training in BMI and data analytics to work in the era of big data; and education in health system improvement science. Learners should be equipped to understand, interpret, and manage both the capabilities and limitations of AI^107^; as well as to implement and evaluate constantly evolving, diverse technology-based interventions^108,109^.

In recognition of the evolving competencies required in digital health care models, the American Medical Association and the Association of American Medical Colleges have advocated for the integration of data science education into the training of HCPs and health administrators^109–111^. While framed within the context of modern digital health, this advocacy is not new^112^. Calls for clinician education to expand beyond it’s bioscience foundation and include systems engineering and information science to mitigate against technical obsolescence dates back over half a century^113,114^. Despite continued expert recommendations, consensus curriculum requirements at the level of the ACGME, Liaison Committee on Medical Education, or National Board of Medical Examiners have yet to reflect significant change^115,116^. More significant progress has been achieved within individual medical schools and health systems, with programmatic development to integrate foundational clinical informatics and digital health knowledge into medical school^117,118^ and residency curriculums^119^, as well as nursing^120^ and healthcare executive continued education^121,122^. Notable institutions, such as *McWilliams School of Biomedical Informatics* at UT Houston and *Jacobs Technion-Cornell Institute* at Cornell Tech, have introduced distinct but equally broad masters curricula aimed at cultivating the diverse expertise required for healthcare innovation and transformation^107,123^. In acknowledgement of current variability, the creation of a Master of Digital Health, analogous to the masters in public health, has recently been proposed^124^. This degress would offer a standardized core curriculum spanning foundational competencies across requisite domains, with optional specialized tracks tailored to specific areas of practice and expertise.

## Towards financial viability—strategies to operationalize the LHS

Wide-spread adoption of the LHS has been constrained, in part by the perceived imbalance between cost and benefit within a predominantly fee-for-service U.S. healthcare model. This challenge is further compounded by dramatically increasing administrative and traditional research costs over recent decades^125–127^. The recent announcement to immediately standardize indirect cost rates of federal research grants will likely compound these challenges for academic institutions, at least in the short- to medium-term^66,67^. Some sociotechnical elements of the LHS, such as HIT–BMI integration, interdisciplinary alignment, workforce training curriculum changes, and QI culture refinement of IRB boundaries, are relatively cost-neutral, primarily requiring continuous cultural prioritization and incentive restructuring to yield long-term impact. In contrast, investments in digital health innovations and the expansion of HIT/BMI infrastructure necessary to support both the LHS and the AI-enabled future of healthcare are substantially cost-additive, particularly when viewed through the lens of traditional research funding models.

At a meso-level, increased internal front-end investments in pragmatic translational, clinical, health services, and health economics research infrastructure may yield downstream economic returns for individual health systems. Such infrastructure enables the evaluations of digital care innovations through rapid-cycle science, generating time-sensitive knowledge to inform superior investment strategy in a competitive environment characterized by rapidly evolving and high-cost health technologies^47^. Organizations with the capabilities and capacity for continuous, data-driven decisions regarding investment in care model innovation, grounded in locally curated knowledge of real-world impact, may gain a meaningful market advantage. This includes confirming that technology-based interventions produce sustained improvements in patient-centered outcomes, determining cost effectiveness at scale, and verifying that AI systems improve operational process efficiency as intended. Examples of health systems deliberately integrating LHS and executive strategy include Penn Medicine’s *Center for Health Care Innovation and Transformation*, University of Colorado’s *Health Innovation Center*, UC San Diego*’s Jacobs Center for Health Innovation*, and Stanford University’s *Clinical Excellence Research Center*^128–133^. These institutions leverage strategic leadership roles, academic infrastructure, multidisciplinary research expertise, and large, high-quality datasets to rapidly identify, evaluate, iterate, and prioritize promising care model interventions and facilitate the implementation and dissemination of these interventions at scale for improved healthcare outcomes, efficiency, and value. Should federal oversight of healthcare AI adopt a decentralized governance model, the burden of infrastructure would fall to individual health systems, effectively creating a new mandatory cost center. This shift could incentivize systems to creatively extract additional local value through investments in LHS infrastructure, as described above. Similarly, thoughtful reallocation of resources from low-value clinical programs towards comparative effectiveness research capabilities could support the development of scalable, evidence-based AI strategies and broader LHS functionality^134,135^.

At a macro-level, planned reductions of indirect cost support from the NIH may increase the financial burden of traditional research, potentially motivating federal and private foundations to expand investments in health services research. Prioritizing scientific inquiry focused on improving care delivery efficiency may serve to accelerate both digital health innovation and the reengineering of care delivery, ultimately enhancing cost effectiveness^136^. There is a growing need for the Centers for Medicare and Medicaid Services to further develop financing and reimbursement models for emerging digital and innovative care models that have yet to be established as standard care^137^. Academic health systems that cultivate the LHS functionality to generate new evidence on the efficacy and cost-effectiveness of novel clinical interventions could be eligible for targeted support. Such support may be especially crucial for publicly funded health systems striving towards value-based care. This may be partly justified if centralization of AI oversight and governance can be achieved at LHS-designated centers and demonstrates lower cumulative costs than similar fragmented processes within every single health system^138^.

Some drivers of rising healthcare costs (e.g., aging population, increasing prevalence of chronic and complex conditions, growing cybersecurity demands) cannot be easily reversed for the subsequent reallocation of capital towards fixed LHS costs, such as HIT–BMI expansion and research infrastructure. However, other causes, such as the dramatic increases in healthcare administration expenses over the past 30 years, warrant targeted interruption to improve system efficiency^125,139,140^. Continued advocacy for insurance regulation reform, including the simplification of prior authorization and related administrative requirements, may help offset the burden of increasingly complex commercial insurer policies^141^. Advances in AI present a pivotal opportunity to reduce costs by semi-automating numerous labor-intensive administrative tasks. Applications include medical coding and billing, insurance authorizations, denial appeals, and other claims processes, and regulatory reporting of quality measures. Early evaluations of such AI-enabled automation are promising, suggesting the potential for significant cost savings through operational efficiency^142–144^.

## Conclusion

**Fig. 1:**
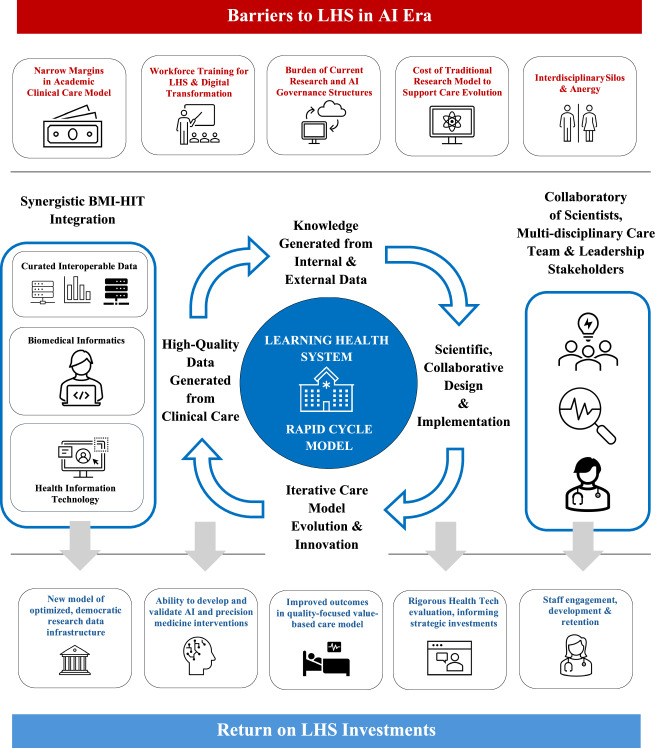
Strategic returns from learning health systems: what overcoming barriers unlocks in the age of AI. By resolving barriers to LHS adoption—ranging from governance inefficiencies to interdisciplinary silos—health systems can unlock transformative returns. These include not only AI-readiness and digital innovation capacity, but also measurable improvements in care quality, operational efficiency, and staff retention.

The LHS affords a compelling model for advancing health care delivery in an era shaped by digital innovation and AI. Although the core principles of the LHS have garnered broad support, realizing their full potential and overcoming challenges requires deliberate investment in data infrastructure, integration of HIT and BMI, workforce development, and adaptive governance (Fig. 1). Foreword thinking academic health systems have demonstrated that meaningful progress is achievable when clinical, operational, and research priorities are aligned around continuous learning. While many health systems face different barriers, creative solutions may enable motivated ones to move forward with this approach (Table 1). Looking ahead, the convergence of AI, digital infrastructure, and operational analytics may enable health systems not only to justify investments in LHS infrastructure but to restructure the cost model of care delivery itself. If AI can meaningfully reduce healthcare’s exorbitant administrative expenses, it may become the first innovation capable of offsetting the cost of its own adoption. Realizing the LHS as a strategic imperative demands more than technical progress—it will require the coordinated redesign of health system infrastructure to enable continuous learning, adaptation, and innovation at scale. Institutions that succeed in this may not only gain a durable competitive advantage but also chart a path toward long-term sustainability in academic health care.

**Table 1:** Catalyzing an AI-ready learning health system: institutional barriers and strategic pathways forward

Barrier	Examples of solutions	Suggested strategy
Disparate biomedical informatics (BMI) and health information technology (HIT) workforce, culture, and strategy.	Multiple examples of ambidextrous HIT–BMI leadership model improving operations, efficiency, and patient-centered outcomes. Importantly, this affords the health system the platform for many of the potential benefits of AI within the LHS paradigm.	Engagement between the health system and academic leadership to highlight the multidimensional benefits of integrating BMI and HIT as the foundation of the LHS within the AI era, highlighting recent successes at large health systems. Identification of specific areas for improvement.
Initial development costs for the infrastructure required to implement digital health interventions, such as clinical AI models	Although short and long-term costs are required for the development and implementation of many clinical AI models, numerous prospective studies demonstrate improvement in patient-centered outcomes.	Engagement with research funding organizations to direct scientific efforts towards determining the clinical utility and cost-effectiveness of mature AI models. Engagement with federal agencies to advocate for “innovation hub” compensation models to mitigate the development costs of generalizable, scalable digital care innovations. Health systems with less technical expertise or capital allocated for innovation may obtain models available in large EHR vendors or from start-up companies. Reallocation of funds from initiatives no longer prioritized or determined effective by the health system may help defray costs related to data storage and cloud computing.
Institutional review boards' interpretation of research ethics may limit rapid cycle testing of Quality Improvement (QI) projects, including the iterative refinements of AI models necessary for successful clinical implementation.	Multiple institutions have successfully developed multi-disciplinary groups that oversee quality improvement initiatives, ensuring that ethics, consent, and privacy standards are met, and AI governance infrastructure.	Engagement with institutions that have successfully utilized quality improvement oversight programs about the necessary steps needed to develop an ethical and patient-protective approach to quality improvement studies, inclusive of IRB exemption, and clinical AI governance.
Cost of appropriately training healthcare workers in LHS and clinical AI concepts.	There are various cost-neutral examples of novel educational curricula training programs that introduce resident and fellow physicians to LHS, digital health, and clinical AI concepts. Any costs may be offset by improved staff engagement, wellbeing, and retention.	Engagement of senior academic leadership to strategically prioritize diverse healthcare worker and healthcare leadership curricula redesign. Will require a collaborative partnership between existing training program leaders, local LHS, and digital health leadership, and national educational entities (e.g., ACGME) Discussion with successful national programs (e.g., PCORI and AHRQ) regarding future expansion of specialist training opportunities. Post-implementation measurement should include staff engagement, wellbeing, and retention: patient-centered outcomes and healthcare delivery costs.
Variable internal and external support for LHS and care innovation (e.g., AI) research.	Multiple examples of academic health systems reorienting care delivery and innovation strategy around science-based evaluation infrastructure and personnel.	Prioritize the recruitment of health services researchers, implementation scientists, and clinical researchers. Work with interested hospital leadership and faculty to promote high-quality LHS initiatives. Emphasis on appropriate mentoring to teach interested individuals at all levels strategies to transform effective ideas into presentations and manuscripts. Advocate for the expansion of and pursue both federal and private foundation funding of health services research. Reallocation of funds from ineffective clinical programs and interventions to support LHS infrastructure and key AI strategies Explore and adopt the use of AI to semi-automate and automate healthcare administration and research tasks.

This table summarizes common challenges to Learning Health System implementation in the context of AI-driven healthcare, with corresponding solutions and strategies to enable scalable, data-informed system transformation.

## Data Availability

No datasets were generated or analysed during the current study.
